# Epidemiological, clinical and therapeutic characteristics of metastatic spinal cord compression in prostate cancer patients in two tertiary hospitals in Cameroon

**DOI:** 10.11604/pamj.2022.41.163.30843

**Published:** 2022-02-23

**Authors:** Etienne Okobalemba Atenguena, Rostand Wilfreed Mbassa Betchem, Anne Marthe Maison Maye, Anne Juliette Sango, Stéphane Zingue, Faustin Dong Zok

**Affiliations:** 1 Department of Internal Medicine, Faculty of Medicine and Biomedical Sciences, University of Yaoundé I, Yaoundé, Cameroon,; 2 Oncology Division, Yaoundé General Hospital, Yaoundé, Cameroon,; 3 School of Health Sciences, Higher Institute of Medical Technology, Yaoundé, Cameroon,; 4 Department of Internal Medicine, Faculty of Medicine and Pharmaceutical Sciences, University of Douala, Douala, Cameroon,; 5 Departement of Internal Medicine, Faculty of Health Sciences, University of Buea, Buea, Cameroon,; 6 Department of Medical and Biomedical Engineering, Higher Technical Teachers´ Training College, University of Yaoundé 1, P.O. Box 886, Yaoundé, Cameroon

**Keywords:** Spinal cord compression, prostate cancer, fracture-settlement, metastatic epiduritis, overall survival

## Abstract

**Introduction:**

prostate cancer represents the 3^rd^ primary neoplasia responsible for metastatic spinal cord compression (MSCC). MSCC is an extreme oncological emergency, because it involves both functional and vital prognosis. The present study aimed to establish a pattern of MSCC in prostate cancer patients in Douala and Yaoundé general hospitals (Cameroon).

**Methods:**

this was a descriptive and retrospective study in the Radiotherapy and Medical Oncology services at both Douala and Yaoundé General Hospitals. The explored variables were general characteristics of the study population, clinical and paraclinical features, management and outcomes. Furthermore 5-year survival was analyzed by the Kaplan-Meier method. Logistic regression by determining the odd ratios and their 95% confidence was done using “Statistical Package for Social Sciences” (SPSS 23) software. The difference was considered significant at p < 0.05.

**Results:**

our series consisted of 151 patients out of which the mean age was 66.88 (SD: 8.71) years (95% CI: 44-88). Pain was the most common clinical symptom (53.33%; n= 80) and fracture-settlement accounted for majority (60%; n= 90.61) of the pain. Thoracic spine damage was encountered by 47.02% (n= 71). Patients received a total doses of irradiation between 20 and 30 gray (Gy). The main toxicity due to radiotherapy were asthenia (45.70%; n= 69.11). The overall survival at 5 years was 90.11%. Factors associated with fracture-settlement were smoking (aOR 10.04, 95% CI: 2.09-48.12; p = 0.004) and the localization of MSCC occurred (aOR 0.21, 95% CI: 0.05-0.77; p = 0.02).

**Conclusion:**

in summary the average age for developing the condition is 66.88 years and factors associated with fracture-settlement were smoking and the localization of MSCC. Back pain was the most common clinical sign and fracture-settlement was the first type of injury on medical magnetic resonance imaging. Therefore, we recommend that emphasis should be placed on increasing awareness of the population on the importance of early diagnosis.

## Introduction

Metastatic spinal cord compression (MSCC) is a compression of the dural sac and its contents (the spinal cord) by an extra-dural tumor mass [1]. It causes pain, muscle weakness progresses into paralysis as well as sensory or sphincter disorders, which not only worsen the survival prognosis but also greatly impair the quality of life. Prostate cancer (10-24%) is the 3^rd^ primary neoplasia after lung (11-35%) and breast (13- 38%) cancers that is responsible for MSCC [2-4]. In fact, these percentages reflect the overall cancer incidences except for cancers of the digestive tract, which are under-represented due to their fewer tendencies to metastasize into the vertebral bodies [5].

Except for malignant melanoma, the multiplicity of MSCC does not appear to be influenced by age, sex or primary tumor type, exception made for malignant melanoma [6,7]. The thoracic vertebrae are the most frequently affected (50-70% cases), followed by the lumbar (20-30%) and cervical (10%) vertebrae [5,6,8]. Some studies have suggested MSCC has the tendency to occur at the lumbosacral level depending on the primary location of the prostate cancer [6,9]. However, this remains controversial [6].

MSCC is an extreme oncological emergency, because it involves not only the functional prognosis but also the vital prognosis. Its incidence in prostate cancer is not precisely known but can still be estimated. In fact, in a series of autopsies, 5-33% of epidural metastases in cancer patients were detected [2,3]. Further, it is estimated that 5-10% of patients with prostate cancer will develop symptomatic MSCC during their lifetime [3,5,6], out of which 15-30% will suffer from multiple MSCC [5-8]. On another hand it has been reported that ~20% of MSCC are the initial manifestation of cancer disease, making it difficult to find the underlying cause, since 78% of cases are due to lung cancer, multiple myeloma, non-Hodgkin's lymphoma or an undetermined origin [10]. In addition, late diagnosis and/or an absence of therapeutic management could lead to paralysis of the lower limbs or even death. The aforementioned therefore raises the issue of how to identify prostate cancer patients with potential MSCC as early as possible. The present study therefore aimed to determine the epidemiological, clinical, and therapeutic characteristics of prostate cancer patients manifesting MSCC, their impact on fractures and main causes of patient death.

## Methods

**Type of study**: this was a descriptive and retrospective study performed by reviewing the digital medical records of prostate cancer patients followed up for MSCC at the Douala and Yaoundé General Hospitals between January 2012 and December 2019 (8 years). The study protocols were approved by the Faculty of Medicine and Pharmaceutical Sciences Institutional Ethical Committee, University of Douala (Cameroon). Patient consent was not required in this retrospective study.

**Presentation of Douala and Yaoundé General Hospitals**: the Douala and Yaoundé General Hospitals are among the most specialized hospitals in Cameroon. Cancer patients followed up in these hospitals are from all parts of the country, which allows a national representation of cancer burden. The Yaoundé General Hospital (YGH) has a number of services specialized in the treatment of cancers such as Radiotherapy, Medical Oncology and Anatomic Pathology, while the Douala General Hospital (DGH) has a Medical Radiotherapy-Oncology Unit equipped with a Cobalt 60 conformational radiotherapy machine. Both Douala and Yaoundé General Hospitals possess experienced practitioners of various specialties related to Medical Oncology.

**Sampling**: our target population consisted of prostate cancer patients with metastatic spinal cord compression (MSCC) registered at the YGH and DGH during the study period (2012-2019). In fact, the inclusion criteria were any patient with histologically proven prostate cancer, suffering from MSCC and followed up either in YGH or in DGH. Patients with MSCC due to other cancers, or with medical record unusable (incomplete or treatment not documented) or not found were excluded. The patients who were included in this study were not at risk and total confidentiality was ensured. A total of 151 patients (98 at DGH and 53 at YGH) were included in this study after an exhaustive recruitment with systematic inclusion of all the medical records of patients who met inclusion criteria in the different services.

**Study design**: the data were collected from patient medical records in the 8 years study period using a pre-established questionnaire as follows: (1) general characteristics (age, marital status, schooling, professional activity, smoking, alcohol, HIV serology, (2) Clinical and Paraclinical data (the reason for consultation, duration of development, date of diagnosis, general condition, associated signs, complications, bone lysis, vertebral collapse, fracture and compression, dislocation, erasure of the vertebral pedicle, (3) management and outcomes (start date, type, protocol used, number of cures, signs of toxicity and complications, progress, end date, the patient's status at the latest news, the date of the latest news). In order to calculate the overall survival at 5 years, the delay of patients within 5 years from the date of diagnosis of MSCC in prostate cancer patients was obtained by active and passive methods. The passive assessment method of survival was based on medical records, while the active method consisted of calling the patients or their family members by phone when the death information were lacking in their medical records. The overall survival at 5 years was calculated by the Kaplan Meier method using SPSS 23 software. Once the data matrix was obtained through Epi Info 7 software, it was transferred to SPSS 23 software for statistical analysis.

**Data analysis**: the descriptive categorical data were reported in percentages and analysis with Pearson's Chi-square statistical test, descriptive numerical data with mean and standard deviation, and logistic regression univariate analysis was performed. P-values less than 0.1 were considered statistically significant to include the retained variables along with their relative modalities in the logistic regression multivariate analysis. The odd ratios were calculated with their 95% confidence interval, in order to assess the influence between the different variables and the fracture-settlement.

## Results

**Figure 1:**
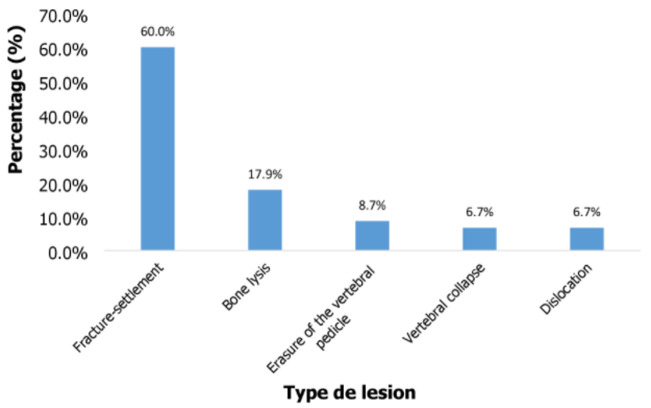
type of lesion recorded after MRI of prostate cancer patients suffering of MSCC

**Fracture-settlements in prostate cancer with MSCC**: in total 172 cases of prostate cancer patients with MSCC were recorded out of which 21 cases were excluded. Fracture-settlements (60%; n= 90.61) which represent the 1^st^ type of lesion recorded after Magnetic Resonance Imaging (MRI) of MSCC was the most common in our study population, followed by bone lysis (18%; n =27.20) and effacement of the vertebral pedicle (8.67%; n = 13.11) (Figure 1).

**Figure 2:**
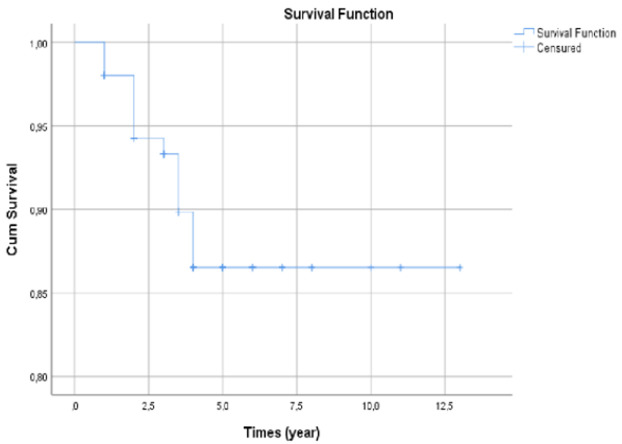
survival curve at 5 years of prostate cancer patients suffering of MSCC

**Survival at 5 years**: patients with prostate cancer and suffering from MSCC had an average survival of 11.62 years (10.92-12.32). The overall survival at 5 years, estimated by the Kaplan Meier method was 90.11% (Figure 2). The majority of patients who were deceased were fractured (11 cases out of 14, 78.11%).

**General and clinical patient characteristics**: Table 1 shows that the average age of prostate cancer patients with MSCC is 66.9 (SD 8.71) years, with the frequency peak in the interval [60;70]. Most of the patients were married (76.51%; 114 out of 149 cases). The most represented religion was Catholic (93 cases out of 150) with 64 fractured cases (68.81%). The fractures were abundantly represented in patients with lower school levels. In terms of frequencies, the study population consisted of 26% (37 cases on 151) alcohol users, 3% (5 cases out of 151) HIV patients and 12% (18 cases out of 151) smokers. Interestingly, all the HIV positive patients were fractured. Four variables out of the seven analyzed were significantly associated with fractures: age, schooling, alcohol and smoking (Table 1). Patients aged > 70 years were 3.67 times (95% CI: 1.02-13.17; p= 0.046) more likely to be fractured than younger ones. Otherwise, patients with post-university school level were less subjected to fracture (aOR 0.29, 95% CI: 0.09-0.89; p = 0.032) than those of the lower level. Alcohol consumption increased the risk of developing fractures by about 2 folds (aOR 1.96, 95% CI: 0.89- 4.33; p = 0.096). Interestingly patients who were smokers had a higher risk (aOR 4.11, 95% CI: 1.49-11.33; p = 0.006) of developing fractures than the non-smoking patients.

**Table 1:** bivariate analysis between characteristics of patients and fracture-settlement

Variables	Total	Fracture	unfractured	p-value
**Age (year)**				**0.081**
60	**27**	20(74.1)	7(25.9)	
60 to 70	**57**	37(64.9)	20 (35.1)	
70 to 80	**52**	44 (84.6)	8 (15.4)	
80	**15**	9 (60)	6 (40)	
**Total**	**150**	110(73.3)	40(26.7)	
**Religion**				0.5
Catholic	**93**	64 (68.8)	29 (31.2)	
Protestant	**35**	27 (77.1)	8 (22.9)	
Islamic	**7**	6 (85.7)	1 (14.3)	
Animist	**2**	2(100)	0(0)	
Others	**13**	11(84.6)	2(15.4)	
**Total**	**150**	110 (73.3)	40 (26.7)	
**Marital status**				0.7
Single	**27**	21 (77.8)	6 (22.2)	
Divorced	**4**	2(50)	2(50)	
Married	**114**	83(72.8)	31(27.2)	
Widower	**4**	3(75)	1(25)	
**Total**	**149**	109 (73.2)	40 (26.8)	
**Schooling**				**0.084**
Primary	**6**	5(83.3)	1(16.7)	
Secondary	**51**	40(78.4)	11(21.6)	
University	**77**	58(75.3)	19(24.7)	
Post-university	**15**	7(46.7)	8(53.3)	
**Total**	**149**	110(73.8)	39(26.2)	
**HIV**				0.32
Yes	**5**	5 (100)	0(0)	
No	**146**	105(71.9)	41(28.1)	
**Total**	**151**	110(72.8)	41(27.2)	
**Alcohol**				**0.093**
Yes	**37**	23(62.2)	14(37.8)	
No	**114**	87 (76.3)	27(23.7)	
**Total**	**151**	110(72.8)	41(27.2)	
**Smoking**				**0.004**
Yes	**18**	8(44.5)	10(55.5)	
No	**133**	102(76.7)	31(23.3)	
**Total**	**151**	110(72.8)	41(27.2)	

**Clinic signs**: Table 2 shows that pain, presented at a rate of 53.33% (80 cases out of 150) in our study population, is the 1^st^ clinical sign of MSCC, followed by paraplegia (31 cases out of 150) and paresthesia (9 cases out of 150). Thoracic vertebrae were the most represented vertebral location of MSCC with a rate of 47.02% (71 cases out of 151), followed by lumbar vertebrae with a rate of 34.44% (52 case out of 151) and cervical vertebrae with a rate of 14.57% (22 cases). In this study it was observed that the risk of fracture increased with the state of the disease. Indeed, patients of state WHO 4 were 5.11 fold (95% CI: 1.43-17.99; p = 0.012) likely to be fractured than those with an acceptable general state. A thoracic localization of MSCC appeared to be the main risk of fracture. Indeed, a cervical localization of MSCC reduced the risk of fracture (aOR 0.35, 95% CI: 0.13-0.96; p= 0.041).

**Table 2:** bivariate analysis between clinical and paraclinical data and fracture-settlement

Variables	Total	Fracture	unfractured	p-value
**Clinical signs**				0.34
Pain	80	63(78.8)	17(21.2)	
Paraplegia	31	21(67.7)	10(32.3)	
Paresthesia	9	3(33.3)	6(66.7)	
Paresia	7	5(71.4)	2(28.6)	
heavy	5	4(80)	1(20)	
Mictional emergency	4	3(75)	1(25)	
Constipation	4	3(75)	1(25)	
Raidor	3	3(100)	0(0)	
Sexual impotence	3	2(66.7)	1(33.3)	
Urinary retention	2	1(50)	1(50)	
Feces incontinence	2	1(50)	1(50)	
**Total**	**150**	109(72.7)	41(27.3)	
**General state of the patient**				**0.04**
WHO1	49	35(71.4)	14(28.6)	
WHO 2	39	31 (79.5)	8(20.5)	
WHO 3	48	38(79.2)	10(20.8)	
WHO 4	14	6(42.8)	8(57.2)	
**Total**	**150**	110(73.3)	40(26.7)	
**T category of TNM classification**				0.4
T 1	17	14 (82.3)	3(17.7)	
T 2	52	40(76.9)	12(23.1)	
T3	53	38(71.7)	15(28.3)	
T 4	26	16(61.5)	10 (38.5)	
**Total**	**148**	108 (73)	40 (27)	
**Level the compression**				**0.089**
Cervical	22	12 (54.5)	10 (45.5)	
Lumbar	52	40 (76.9)	12 (23.1)	
Sacred	6	3 (50)	3 (50)	
Thoracic	71	55 (77.5)	16 (22.5)	
**Total**	**151**	110 (72.8)	41 (27.2)	

**Management and outcomes**: Table 3 depicts that a total of 91 patients (60.31%) were irradiated with a dose of 30 Gy or less in 10 fractions. It is worthwhile to notice that patients irradiated (69 cases, 75.81%) were fractured whatever the dose received. Asthenia was the first toxicity encountered in the irradiated population. Patients treated with high doses of corticotherapy had a higher amount of fractures with a maximum at the dose of 10 mg (16 cases out of 17, 94.12%). Only the total dose of corticosteroid received was included in the logistic regression multivariate analysis. Indeed, patients who received 10 mg were 13 times (aOR 13, 95% CI: 1.52-111.5; p= 0.019) more likely to be fractured than the others.

**Table 3:** bivariate analysis between management and outcomes of prostate cancer patients with MSCC and fracture-settlement

Variables	Total	Fracture	unfractured	p-value
**Total dose of Radiotherapy**				0.35
10 Gy	**43**	31 (72.1)	12 (27.9)	
20 Gy	**17**	10 (58.8)	7 (41.2)	
30 Gy	**91**	69 (75.8)	22 (24.2)	
**Total**	**151**	110 (72.8)	41 (27.2)	
**Toxicity of radiotherapy**				0.3
Amnesia	**7**	4 (57.1)	3 (42.9)	
Asthenia	**69**	52 (75.4)	17 (24.6)	
Headache	**6**	3 (50)	3 (50)	
Difficulty concentrating	**5**	2 (40)	3 (60)	
Irritability	**8**	5 (62.5)	3 (37.5)	
Nausea	**42**	32 (76.2)	10 (23.8)	
radioepithelitis	**14**	12 (85.7)	2(14.3)	
**Total**	**151**	110 (72.8)	41 (27.2)	
**Total dose of corticotherapy**				**0.07**
100 mg	**28**	21 (75)	7 (25)	
24 mg	**67**	50 (74.6)	17 (25.4)	
10 mg	**17**	16 (94.1)	1 (5.9)	
4 mg	**10**	7 (70)	3 (30)	
Others	**29**	16 (55.2)	13 (44.8)	
**Total**	**151**	110 (72.8)	41 (27.2)	
**Toxicity of corticotherapy**				0.36
Confusional state	**7**	5(71.4)	2(28.6)	
Hyperglycemia	**6**	3(50)	3(50)	
Hypomania	**9**	8(88.9)	1(11.1)	
Infection	**3**	3(100)	0(0)	
Proximal myopathy	**26**	20(76.9)	6(23.1)	
Edema	**31**	25(80.6)	6(19.4)	
Psychosis	**8**	4(50)	4(50)	
Peptic ulcer	**61**	42(68.8)	19(31.2)	
**Total**	**151**	110(72.8)	41(27.2)	
**Biphosphonates**				0.203
Zoledronic acid	**135**	99(73.3)	36(26.7)	
Pamidronate	**11**	9(81.8)	2(10.2)	
Others	**5**	2(40)	3(60)	
**Total**	**151**	110(72.8)	41(27.2)	
**State at the last view**				0.61
Deceased	**14**	11(78.6)	3(21.4)	
Survivor	**137**	99(72.3)	38(27.7)	
**Total**	**151**	110(72.8)	41(27.2)	

**Final model of logistic regression multivariate analysis**: seven variables were selected in the final model of our study: age, schooling, alcohol, smoking, state of disease, compression level and total dose of corticotherapy. Significant association with fracture-settlements was found with smoking (aOR 10.04, 95% CI: 2.09 - 48.12; p = 0.004) and the localization of the MSCC. MSCC occurring in the cervical region presented less risk (aOR 0.21, 95% CI: 0.05 - 0.77; p = 0.02) of developing a fracture than when it happened in the thoracic region (Table 4).

**Table 4:** linear regression multivariate analysis of factors associated with fracture-settlement

Variables	Univariable analysis		Multivariable analysis	
	OR [95% CI]	p-value	OR [95% CI]	p-value
**Age (year)**		**0.093**		0.197
80	1		1	
60	1.91[0.50 7.31]	0.348	1.71[0.29 9.72]	0.557
60 to 70	1.23 [0.38 3.96]	0.725	1.34 [0.29 6.22]	0.708
70 to 80	3.67 [1.02 13.17]	0.046	4.32[0.80 23.40]	0.089
**Schooling**		0.111		0.155
University	1		1	
Post-university	0.29[0.09 0.89]	0.032	0.18 [0.04 0.84]	0.029
Primary	1.63 [0.18 14.9]	0.661	0.90 [0.89 9.81]	0.926
Secondary	1.19[0.51 2.77]	0.685	0.98[0.35 2.72]	0.967
**Alcohol**		**0.096**		0.561
No	1		1	
Yes	1.96[0.89 4.33]	0.096	0.69 [0.19 2.44]	0.561
**Smoking**		**0.066**		**0.004**
No	1		1	
Yes	4.11 [1.49 11.32]	0.006	10.04[2.09 48.12]	0.004
**State of disease**		**0.059**		0.496
WHO4	1		1	
WHO 1	3.33[0.98 11.37]	0.054	2.46[0.44 13.82]	0.307
WHO 2	5.17[1.39 19.21]	0.014	4.03[0.68 23.97]	0.126
WHO 3	5.11[1.43 17.99]	0.012	2.51[0.42 14.91]	0.313
**Compression level**		0.104		**0.031**
Thoracic	1		1	
Cervical	0.35[0.13 0.96]	0.041	0.21[0.05 0.77]	0.02
Lumbar	0.97 [0.41 2.27]	0.944	1.73 [0.56 5.35]	0.339
Sacred	0.29[0.05 1.58]	0.153	0.56[0.72 4.37]	0.581
**Total dose of corticotherapy**		0.118		0.173
Others	1		1	
100 mg	2.45[0.79 7.51]	0.121	1.76[0.44 7.01]	0.425
10 mg	13.0 [1.52 111.5]	0.019	23.1[1.8 296.7]	0.016
24mg	2.40[0.96 5.97]	0.062	2.06[0.62 6.82]	0.236
4 mg	1.90 [0.41 8.82]	0.415	1.07[0.16 6.92]	0.944

## Discussion

The present study aimed to determine the epidemiological, clinical, and therapeutic characteristics of prostate cancer patients manifesting MSCC, their impact on fractures and main causes of patient death. It was found that the mean age of patients suffering from prostate cancer and MSCC was 66.88 (SD 8.71) years with a minimum of 44 years and a maximum of 88 years. This value is close to the findings of Hoskin *et al*. [11] who showed that 102 patients suffering from MSCC confirmed by MRI had a median age of 68 years. The works of Djiencheu *et al*. [12] and Engbang *et al*. [13] performed in Cameroon showed an average age of 52 years and 55.8 ± 15.41 years with extremes at 12 and 84 years, respectively, which deviates a little from the values of this study. This difference could be due to the fact that in these studies the MSCC were from any type of cancer in women and men. The insidious nature of tumor growth may also be another explanation. Pain was the first clinical sign, affecting 53.3% of the study population. MSCC can occur in several forms, but in most of the cases, pain is found. This result is in accordance with that of Gilbert *et al*. [4] who found pain as the first clinical sign in 96% of cases. In addition, Engbang *et al*. also recorded pain in 98.4% of cases with 66.4% lumbar pain, 28.4% chest pain and 5.2% neck pain. This can be explained by the fact that back pain is one of the telltale signs of spinal cord compressions. It is therefore important in the presence of back pain to rule out the hypothesis of probable MSCC for better support and management of MSCC.

Fracture-settlement was the 1^st^ type of vertebral bone lesion on the MRI of patients with MSCC and it accounted for MSCC with 60% of cases. This observation corroborates the findings of Metoui *et al*. [14], who showed a vertebral settlement of the L3 vertebra with medullar compression. It is therefore up to the medical oncologist to think of an MSCC in the face of any spinal cord compression. The thoracic vertebrae were the most affected vertebral location with a proportion of 44.02% that could cause spinal cord damage. This predominance (observed in 50-70%) was also reported by a number of authors [5,6,8,15]. In addition, Deborah *et al*. [16] showed that spinal cord damage is most often produced in the thoracic region in 95% of patients presenting with MSCC. However, other studies have rather shown lumbosacral localization as the most frequent localization [5,9]. In previous studies performed by Enbang *et al*. [13] in Cameroon, the main location of MSCC was in the lumbar (57.1%), thoracic (37.7%) and cervical (5.2%) vertebrae.

It was found in this study that 26% of the patients regularly consumed alcohol and 12% were smokers. These results show that there is no real association between alcohol and MSCC, while smoking greatly affects the occurrence of MSCC in patients suffering from prostate cancer. These results are in line with those of Silva *et al*. [17] who showed a strong association between MSCC in patients who smoked and were suffering from lung cancer. The total dose of radiotherapy received in our population was 30 Gy delivered in 10 fractions in 91 cases out of the 151 irradiated. Of note, irradiation of the spine is well known to reduce back pain and an improve the ability to walk. This observation is in line with many reports [1,2,8]. Moreover, the results published by Maranzano *et al*. [8], showed that a high dose leads to greater tumor destruction. This therapeutic modality has to be taken into account in the management of patients with metastatic spinal cord compressions. It was found in this study that 67 patients out of 151 received a dose of 24 mg of corticosteroid therapy. These results corroborate those of Vecht *et al*. [18] who reported the dose of 10 to 100 mg of dexamethasone. This can be explained by the fact that breast cancer is the most common cancer in women and prostate cancer the most common in men.

Bone is a prime site of metastasis for many solid tumors, and the complications associated with bone metastases can lead to significant bone morbidity, including MSCC. The most common primary cancer was prostate cancer (43.2%), followed by breast (23.2%). These results are similar to several studies including those preformed in Cameroon [5,12,13]. The overall survival at 5 years from diagnosis was 90.11% with the survival of 11.62 months (10.92- 12.32); suggesting a poor lethality. This observation is in line with many others who showed that the overall survival after diagnosis of MSCC is between 6-9 months with 28% survival at 1 year [2,8]. In this study, we faced the common limitations of a retrospective study, including missing data, large proportion of loss to follow up, which resulted in numerous unrecovered medical records. Faced with these obstacles, we excluded 21 files with missing data, we ignored 15 files that could not be found, and we focused our work on the 151 remaining files. However, the results presented in this study give an idea of the overall survival at 5-years of prostate cancer patients suffering from MSCC in Cameroon and the main factors that influence the fracture-settlement.

## Conclusion

The metastatic spinal cord compression in prostate cancer is an emergency health condition in oncology. The average age for developing the condition is 66.88 years. Back pain was the most common clinical sign. Fracture-settlement was the first type of injury on medical magnetic resonance imaging. The thoracic vertebrae were the most represented vertebral location. The combination of radiotherapy and corticosteroid therapy was the treatment modality. Asthenia was the main complication of radiotherapy and the overall survival of the general population was 90.11% at 5 years. Factors associated with fracture-settlement were smoking and the localization of MSCC. We recommend that emphasis should be placed on increasing awareness of the importance early diagnosis in the general population.

### What is known about this topic

- It is well known that MSCC is an extreme oncological emergency, involving both functional and vital prognosis;
- Back pain is the most common clinical sign of MSCC, Fracture-settlement was the first type of injury on medical magnetic resonance imaging;
- The overall survival at 5 years in Cameroon was 6-9 months with 28% survival at 1 year.

### What this study adds

- This is a new study showing the recent pattern of prostate cancer patients suffering from MSCC;
- Two factors were associated with fracture-settlement in patient with MSCC: smoking and the localization of the MSCC;
- Patients with prostate cancer and suffering from MSCC had an average survival of 11.62 years (10.92 - 12.32). The overall survival at 5 years was 90.11%.

## References

[1] Loblaw DA, Lapierre NJ (1998). Emergency treatment of malignant spinal cord compression: an evidence-based guideline. J Clin Oncol.

[2] Kovner F, Spigel S, Rider I, Otremsky I, Ron I, Shohat E (1999). Radiation therapy of metastatic spinal cord compression: multidisciplinary team diagnosis and treatment. J Neuro-Oncol.

[3] Zaidat OO, Ruff RL (2002). Treatment of spinal epidural metastasis improves patient survival and functional state. Neurology.

[4] Tatsui H, Onomura T, Morishita S, Oketa M, Inoue T (1996). Survival rates of patients with metastatic spinal cancer after scintigraphic detection of abnormal radioactive accumulation. Spine.

[5] Gilbert RW, Kim JH, Posner JB (1978). Epidural spinal cord compression from metastatic tumor: diagnosis and treatment. Ann Neurol.

[6] Schiff D, O´Neill BP, Wang CH, O´Fallon JR (1998). Neuroimaging and treatment implications of patients with multiple epidural spinal metastases. Cancer.

[7] van der Sande JJ, Kröger R, Boogerd W (1990). multiple spinal epidural metastases: an unexpectedly frequent finding. J Neurol, Neurosurg, Psychiatry.

[8] Maranzano E, Latini P (1995). Effectiveness of radiation therapy without surgery in metastatic spinal cord compression: final results from a prospective trial. Int J RadiatOncolBiolPhys.

[9] Flounders JA, Ott BB (2003). Oncology nursing modules: spinal cord compression. ONF.

[10] Schiff D, O´Neill BP, Suman VJ (1997). Spinal epidural metastasis as the initial manifestation of malingnancy: clinical features and diagnostic approach. Neurology.

[11] Hoskin PJ, Grover A, Bhana R (2003). Metastatic spinal cord compression: radiotherapy outcome and dose fractionation. radiotherapy and oncology.

[12] Djiencheu Njamnshi Ngandeu (2007). compressions médullaires lentes d´origines tumorale et pseudo-tumorale à yaounde. AFJ neurol sci.

[13] Engbang JP, Motah M, Essola B, Metso L (2021). Epidemiological, clinical, diagnostical and histopathological aspects of medular compressions of metastatic origin. Cancer Research Journal.

[14] Leila Metoui, Faïda Ajili, Mouna Maiza, Mehdi Ben Ammar, Imen Gharsallah, Issam M'sakni (2013). Spinal cord compression an intra osseous schannoma. Case reports in medicine 2013.

[15] Per Soelberg Sørensen, Svend Erik Børgesen, Karen Rohde, Bente Rasmusson, Flemming Bach, Torben Bøge-Rasmussen (1990). Metastatic epidural spinal cord compression: results of treatment and survival. Cancer.

[16] Deborah A, Anas M, Paul F, Sture V (1986). Characteristics of spinal cord compression in adenocarcinoma of prostate. Urology.

[17] Silva GT, Anke B, Luiz CST (2015). Incidence, associated factors and survival in metastatic compression of the spinal cord secondary to lung cancer. the spine journal.

[18] Vecht ChJ, Haaxma-Reiche H, Van Putten W (1989). Initial bolus of conventional versus high-dose dexamethasone in metastatic spinal cord compression. Neurology.

